# Feeding of cows’ milk formula at maternity hospital and the development of cows’ milk allergy

**DOI:** 10.1186/2045-7022-1-S1-P102

**Published:** 2011-08-12

**Authors:** Stefano Mazzoleni

**Affiliations:** 1 Aulss 16 Regione Veneto, Padova, Italy

## Background

Early feeding with cows' milk (CM) formula may cause CM allergy (CMA). Contribution of CM formula feeding at maternity hospital in the development of CMA was studied.

## Methods

27 children with CMA were retrospectively examined in this study: 12 boys and 15 girls, the median age at the beginning of symptoms was 5 months (range 18 days - 6 years). In 18 children symptoms began quickly after ingesting CM (urticaria and angioedema 8 cases, anaphylaxis 7, skin rash 2, vomit 1 case); in 9 children symptoms began several hours after. All children performed skin prick test and food challenge test.

## Results

In 12 out of 27 patients (44%) symptoms of CMA appeared after ingestion of the "first" meal of CM formula or dairy products at home. Children with rapid reaction had symptoms after the "first" meal of CM in 66% of cases. A retrospective examination of neonatal charts revealed that nine out of 12 children who reacted to the “first” meal of CM at home had received CM formula at the hospital and were fully breastfed after discharge. Only two out of 12 of these children was born to atopic parents.

## Conclusions

In our experience feeding of CM formula at the maternity hospital is involved in about one-third of CM allergy. Avoidance of bottles during the establishmet of breastfeeding may give a relevant contribution to the prevention of CMA in children. This measure should be applied to all neonates from atopic and non-atopic parents.

